# Gamma camera characterization at high holmium-166 activity in liver radioembolization

**DOI:** 10.1186/s40658-021-00372-9

**Published:** 2021-03-02

**Authors:** Martina Stella, Arthur J. A. T. Braat, Marnix G. E. H. Lam, Hugo W. A. M. de Jong, Rob van Rooij

**Affiliations:** Department of Radiology and Nuclear Medicine, UMC Utrecht, Heidelberglaan 100, 3584 CX Utrecht, The Netherlands

**Keywords:** Radioembolization, ^166^Holmium, High-count rate, Scatter correction, Dosimetry

## Abstract

**Background:**

High activities of holmium-166 (^166^Ho)–labeled microspheres are used for therapeutic radioembolization, ideally directly followed by SPECT imaging for dosimetry purposes. The resulting high-count rate potentially impacts dead time, affecting the image quality and dosimetric accuracy. This study assesses gamma camera performance and SPECT image quality at high ^166^Ho activities of several GBq. To this purpose, the liver compartment, including two tumors, of an anthropomorphic phantom was filled with ^166^Ho-chloride, with a tumor to non-tumorous liver activity concentration ratio of 10:1. Multiple SPECT/CT scans were acquired over a range of activities up to 2.7 GBq. Images were reconstructed using a commercially available protocol incorporating attenuation and scatter correction. Dead time effects were assessed from the observed count rate in the photopeak (81 keV, 15% width) and upper scatter (118 keV, 12% width) window. Post reconstruction, each image was scaled with an individual conversion factor to match the known total activity in the phantom at scanning time. The resulting activity concentration was measured in the tumors and non-tumorous liver. The image quality as a function of activity was assessed by a visual check of the absence of artifacts by a nuclear medicine physician. The apparent lung shunt fraction (nonzero due to scatter) was estimated on planar and SPECT images.

**Results:**

A 20% count loss due to dead time was observed around 0.7 GBq in the photopeak window. Independent of the count losses, the measured activity concentration was up to 100% of the real value for non-tumorous liver, when reconstructions were normalized to the known activity at scanning time. However, for tumor spheres, activity concentration recovery was ~80% at the lowest activity, decreasing with increasing activity in the phantom. Measured lung shunt fractions were relatively constant over the considered activity range.

**Conclusions:**

At high ^166^Ho count rate, all images, visually assessed, presented no artifacts, even at considerable dead time losses. A quantitative evaluation revealed the possibility of reliable dosimetry within the healthy liver, as long as a post-reconstruction scaling to scanning activity is applied. Reliable tumor dosimetry, instead, remained hampered by the dead time.

## Background

Holmium-166 (^166^Ho) radioembolization (QuiremSpheres®, Quirem Medical B.V., Deventer, The Netherlands) is a well-established treatment for primary and secondary tumors in the liver [1]. This procedure consists of injecting radioactive microspheres via a microcatheter in the hepatic artery. The microspheres preferentially lodge in the tumors’ arterioles and ^166^Ho, being a beta emitter, irradiates the tumor cells, sparing the healthy liver. In ^166^Ho radioembolization, single photon emission computed tomography (SPECT)/CT is used for dosimetry purposes both for treatment planning and post-treatment evaluation, relying on the additional 80.6 keV gamma emission of ^166^Ho. In the pre-treatment phase, the distribution of low activity ^166^Ho-microspheres (i.e., scout dose, 250 MBq, QuiremScout®, Quirem Medical B.V., Deventer, The Netherlands) can be directly translated to microsphere distribution at therapeutic levels and can be used to identify potential extrahepatic depositions [2]. In the post-treatment phase, the actual therapeutic activity distribution is assessed to establish a dose response relation or to verify whether a sufficient absorbed dose was delivered to tumor tissue in individual cases. When using ^166^Ho-microspheres, approximately 3.8 GBq of ^166^Ho is injected per kilogram of treated liver mass [3] in order to deliver 60 Gy to the injected liver portion, as indicated by the manufacturer, and assuming a homogenous uptake throughout the liver. This typically results in injected activities of 5–10 GBq. Although the abundancy of the 80.6 keV ^166^Ho photopeak is only 6.7%, the count rate immediately after treatment is very high. This is a consequence of the occurrence of multiple gamma emissions in the MeV range (1.38 MeV/0.93%, 1.58 MeV/0.19%, 1.66 MeV/0.12%) of the ^166^Ho spectrum, causing interactions within the patient, collimator, and crystal. Furthermore, there is a considerable amount of bremsstrahlung, caused by β^−^ (E_max_:1.77 Mev/49%, 1.85MeV/50%) interactions within the patient [4, 5]. This potentially impacts image quality and quantitative accuracy because of gamma camera dead time effects. For accurate post-treatment dosimetry, a time interval between ^166^Ho treatment and image acquisition is therefore used in clinical practice, which, given the 26.8 h half-life, is typically 3–5 days post-treatment. However, this may be undesirable from a logistical perspective.

This study aimed to assess SPECT image quality for a range of ^166^Ho activities, from 447 MBq extending up to 2.7 GBq (equivalent to a treatment-imaging interval of approximately two days in a clinical scenario). In addition, the possibility to perform relative dosimetry within healthy liver and tumors, at different activity concentrations, was investigated.

In a preliminary study [4], Elschot et al. evaluated scintillation camera characteristics for isotopes used in liver radioembolization, including ^166^Ho. To this purpose, a NEMA image quality phantom was used, and a comparison between isotopes using various collimators, to optimize acquisition parameters and to support future analyses of clinical comparisons between radioembolization studies was systematically performed. Additionally, the count rate linearity of the camera (Philips FORTE) was assessed. However, the count rate linearity was determined indirectly, combining the measured system sensitivity with an *intrinsic* count rate curve (i.e., measured on a spectrum without patient Compton scatter nor collimator interactions).

In this study, a further investigation mimicking patient geometry and clinical protocol for image acquisition and reconstruction is presented, including the effect of patient scatter and pulse-pile up at high-count rate. The outcome of this analysis may be used to further optimize imaging protocols and to create guidelines for ^166^Ho SPECT acquisition in clinical practice, especially for the purpose of quantitative SPECT imaging for post-treatment dosimetry.

## Methods

To estimate the effect of dead time and count pile-up, count rate performance and measured activity concentration were assessed, considering the total activity at scanning time. The image quality was evaluated by a nuclear medicine expert (experience > 5 years) who visually checked the absence of image artifacts. Measured lung shunt fractions (LSF_measured_) for the considered range of activities were determined on both planar and SPECT acquisitions. Activity in the phantom was limited to 2.7 GBq to limit radiation dose to the investigators (due to filling and frequent handling of the phantom).

### Phantom characteristics

**Fig. 1:**
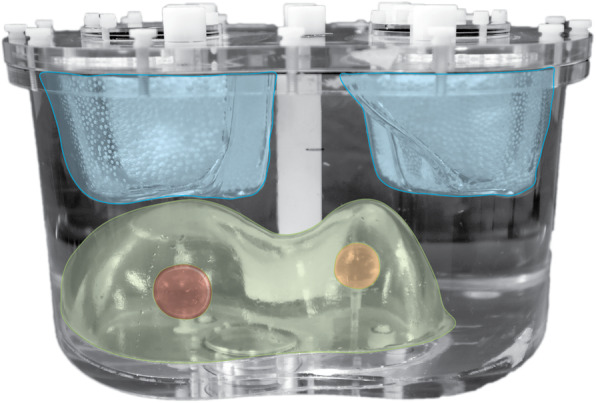
Anthropomorphic phantom model ECT/TOR/P used for the experiment. A photograph of the phantom with VOIs superimposed. Lung region is colored in light blue, liver compartment in green, sphere S1 in red, and S2 in orange

An anthropomorphic phantom (model ECT/TOR/P), including the lungs and liver, was used to mimic patient anatomy (Fig. 1). Two spheres (S1 and S2) were placed in the liver to resemble tumors of different sizes. The liver compartment (1205 mL) and spheres (S1: volume = 24.2 mL, radius = 1.79 cm and S2: volume = 15.7 mL and radius = 1.55 cm) were filled with ^166^Ho-chloride with a tumor to non-tumorous liver activity concentration ratio of 10:1, resembling a high tumor-to-non-tumor uptake, typically reported for large, highly vascularized tumors [6, 7].

This resulted in an activity percentage, with respect to the total activity in the liver, equal to 15.10% for S1 and 9.76% for S2.

By letting the activity decay, multiple SPECT/CTs were acquired over several days to obtain a range of activities from 447 MBq up to 2.7 GBq. No radioactivity was injected into the lung compartment of the phantom.

### Data acquisition

A total of 16 measurements were performed. All images were obtained using Symbia SPECT/CT scanners (Siemens, Erlangen, Germany), using medium-energy collimators. Of the 16 measurements, 10 were acquired on a Symbia T16 scanner and 6 on a Symbia T scanner. Projections were recorded in the 81 keV (15% width) photopeak window on a 128 × 128 matrix (pixel spacing, 4.8 × 4.8 mm), with 120 angles (20 s per projection) over a non-circular 360^o^ orbit using step-and-shoot mode. An additional energy window centered at 118 keV (12% width) was used to correct the ^166^Ho photopeak data for scatter from higher energy gamma emissions and bremsstrahlung using a window-based scatter correction.

**Fig. 2:**
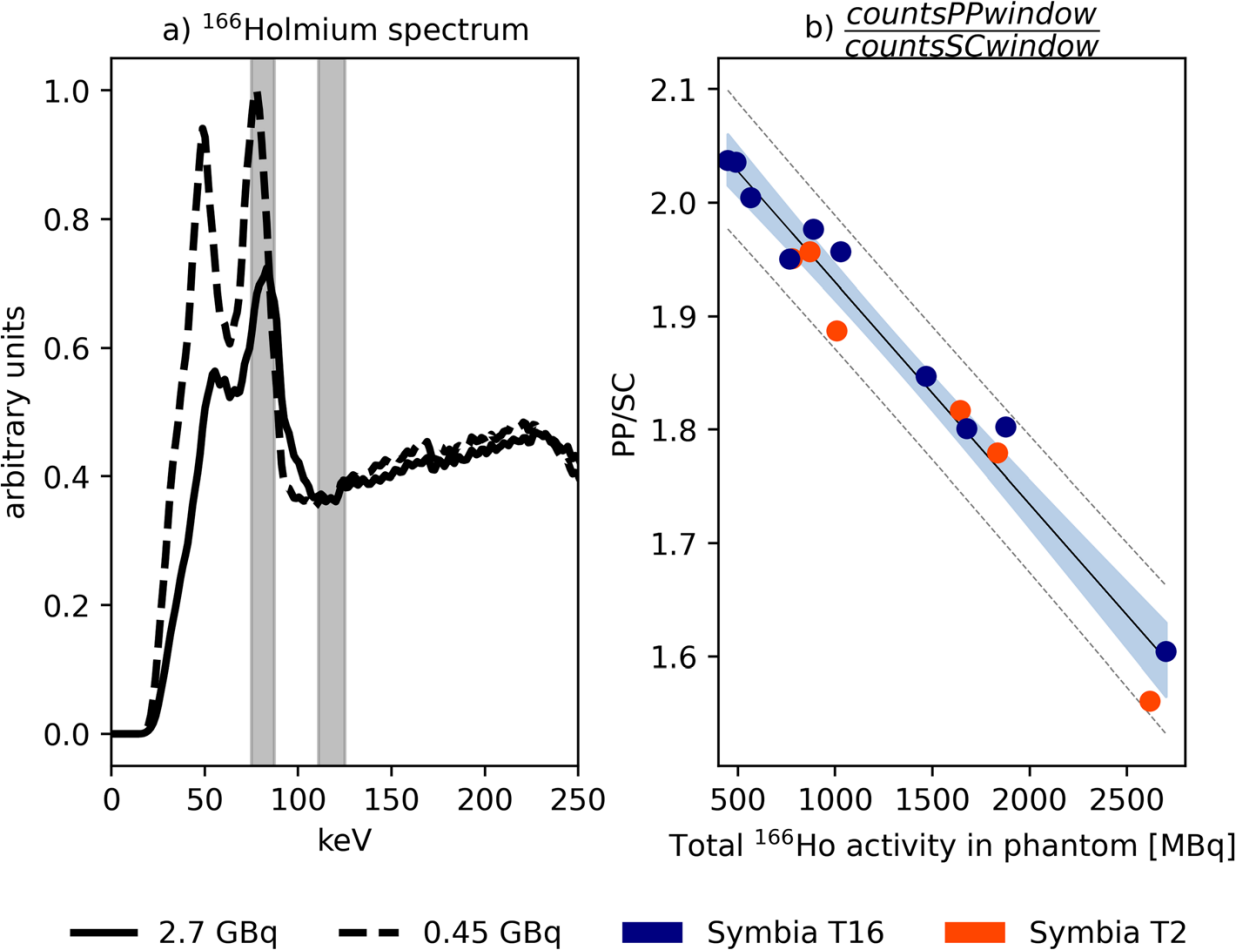
**a** ^166^Ho spectrum acquired for dead times equal to 42.25% (solid line), corresponding to a total phantom activity of 2.7 GBq and 9.25% (dashed line), corresponding to 447 MBq. The curves were scaled for comparison by matching the sum of the counts in the scatter window centered at 118 keV (12% width). The main ^166^Ho photopeak window (80.6 keV, 15% width) and the scatter window (118 keV, 12% width) are represented by the gray areas. The effect of pulse pile-up due to the high-count rate is visible as the shifting and broadening of the photopeak**. b** The ratio between the mean of counts in the 81 keV photopeak window and the 118 keV upper scatter window within the recorded spectra, plotted as function of total activity in the phantom at time of recording. Linear regression is represented by the black line, while gray dashed lines represent prediction intervals. Data in the blue area are within the confidence intervals

The imaging protocol was adopted from the clinical protocol used in patients after a radioembolization treatment. Prior to every measurement, the energy spectrum as recorded by the gamma camera was stored (Fig. 2a).

Before SPECT scans, planar images of the lungs and liver area were acquired (5-min acquisition, 256 × 256 matrix), according to the protocol currently in use for patients. The detectors were positioned to acquire anterior and posterior images. Data were acquired in a 74.6–86.6 keV energy window with the medium-energy low penetration collimators mounted.

### Data reconstruction

Images were reconstructed using commercially available software (Symbia Flash 3D), with 10 iterations, 8 subsets, incorporating scatter and attenuation correction. No Gaussian smoothing was applied. The total downscatter estimate in the 81 keV window included scattered photons originating from higher energy emissions (including bremsstrahlung from the β− emissions), but not the photopeak scatter (originating from the primary 81 keV photopeak). This contribution can be estimated from the 118 keV window, using the k-factor. The k-factor used to correct the photopeak window for downscatter was previously estimated [8, 9] and adopted in this study.

No scatter correction was applied to planar acquisitions.

### Data analysis

**Fig. 3:**
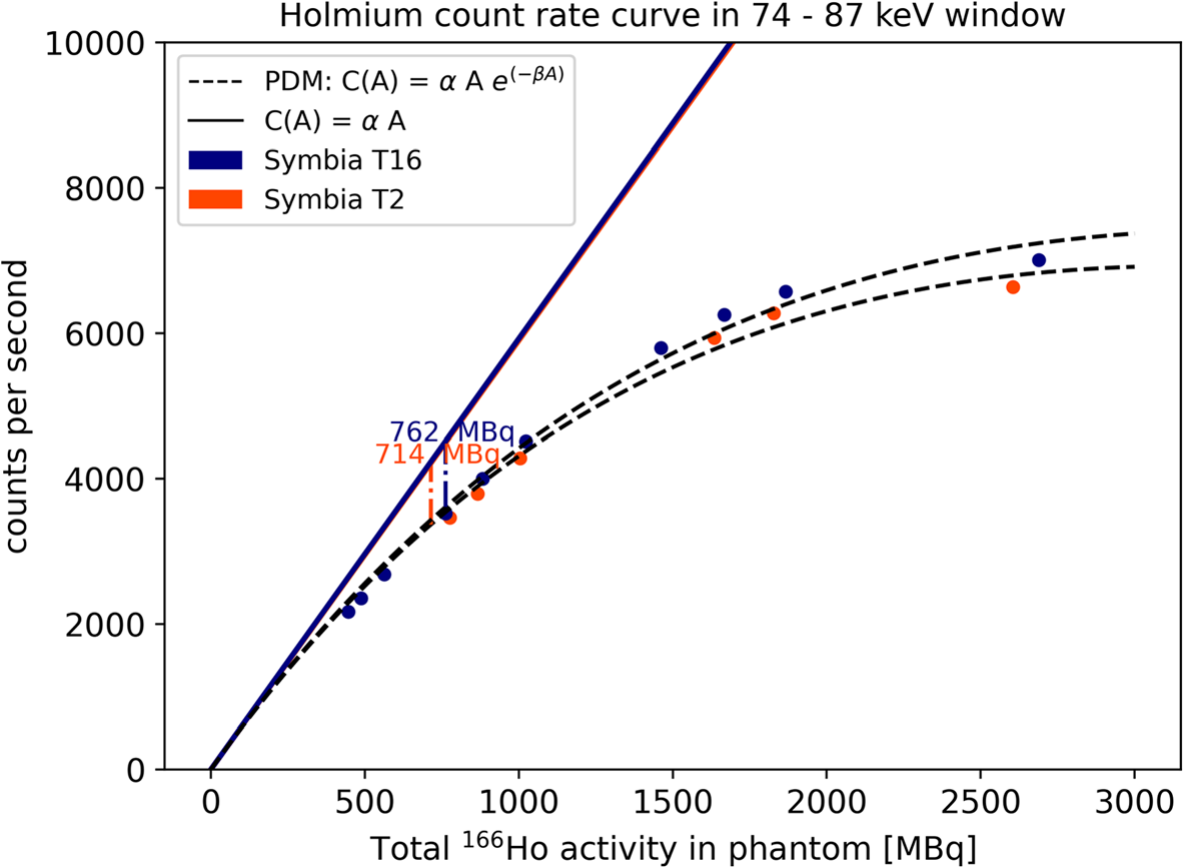
Count rate measured on a Siemens Symbia T16 and T2. Linear model, C(A) = αA is depicted by the solid lines and the paralyzable dead time model (C(A) = αAe^-βA^) by dashed lines. Values for 20% count loss are reported: 714 MBq for Symbia T2 and 762 MBq for Symbia T16

To characterize the gamma camera count-rate performance, counts in the ^166^Ho photopeak window were plotted against total activity in the phantom at scanning time (Fig. 3). The paralyzable detector model (PDM), valid within the considered count range [10], was fitted to the data and forced to pass through the axis origin, to obtain the linear response coefficient and activity at 20% count loss.

Reconstructions were converted into units of Bq by applying a conversion factor based on the known activity in the phantom at the time of scanning. For every reconstruction, a conversion factor was determined by summing the values within a VOI encompassing the liver, enlarged by 2 cm to compensate for spill-out (partial volume effect), and dividing by the activity in the phantom.

The quality of the reconstructed images was assessed visually, by a nuclear medicine physician, and quantitatively evaluating the measured activity concentrations and LSF_measured_. Multiple volumes of interest were defined for the two spheres (S1 = 24.2 mL and S2 = 15.7 mL) within the liver, the liver compartment, and the lungs. The VOIs for the liver and lungs were semi-automatically delineated on the CT images, using ITK-SNAP [11], and subsequently registered onto the corresponding SPECT images using the affine transformation between both coordinate systems. The spheres were instead defined on the SPECT reconstruction as spherical VOIs with radius corresponding to the nominal spheres radius enlarged by 1 cm, to compensate for the partial volume effect (PVE). The size of the sphere radius enlargement was empirically determined by iterative increments of radius size until the effect of PVE was mostly compensated. At this amount of sphere radius dilation, approximately 25% of the activity in the sphere VOI is due to the background, which is corrected for, as shown in Eq. (1). This ensures that most of the activity recovered in the spheres is actually present within the nominal sphere’s volume.

Finally, to obtain the non-tumor VOI (non-tumorous liver) mask, the dilated sphere VOIs were subtracted from the liver VOI, eroded by 1 cm to compensate for the spill-out near the edge.

To assess the possibility of dosimetry in the considered compartments, tumorous and non-tumorous liver, at high-count rate, activity concentration recovery (ACR) was considered.

ACR for spheres was computed as follows:

1 $$ A{CR}_{sphere}=\frac{C_s}{C_{ns}}x\ 100\%=\frac{\frac{A_d}{V_s}-\frac{C_b\left({V}_d-{V}_s\right)}{V_s}}{C_{ns}}\ x\ 100\% $$

where *C*_*s*_ is the measured activity concentration in the sphere, *C*_*ns*_ the nominal activity concentration in the sphere, *V*_*s*_ the nominal sphere’s volume, *A*_*d*_ the measured activity in the dilated sphere, *V*_*d*_ the volume of the dilated sphere, and *C*_*b*_ the measured the activity concentration in the background. The term $\frac{C_b\left({V}_d-{V}_s\right)}{V_s}$ compensates for the recovered activity in the dilated sphere VOI due to activity background.

For the non-tumorous liver background compartment, ACR was computed as follows:

2 $$ A{CR}_{liver\ background}=\frac{C_{lb}}{C_{nl}}x\ 100\% $$

where *C*_*lb*_ is the measured activity concentration and *C*_*nl*_ the nominal activity concentration in the liver background.

The Spearman rank correlation coefficient (*ρ*) between the measured activity concentration and total activity in the phantom at scanning time was computed for each VOI: non-tumorous liver, S1 and S2.

To assess the LSF_measured_, two approaches were used: planar imaging-based and SPECT-based. For both approaches, the scatter in the lungs was computed, in line with clinical practice, as follows:

3 $$ {LSF}_{measured}=\frac{counts_{Lungs}}{counts_{Lungs}+{counts}_{Liver}}\ x\ 100\% $$

For the planar-based method, the counts were computed using the geometric mean following standard clinical practice [12], while for the SPECT-based method, counts within the lung and liver VOIs were considered. Since the lung compartment of the phantom was not filled with any activity, the nominal lung shunt fraction, LSF_nominal,_ was 0 across the range of considered activities.

To visually assess image quality, a nuclear medicine physician (AB, experience >5 years) was randomly and blindly presented the reconstructed images, and asked to visually check the absence of image artifacts.

## Results

Figure 2a depicts the measured ^166^Ho spectrum, acquired at 2.7 GBq and 447 MBq, indicating the transformation of the measured spectra due to pulse pile-up. The dead-time percentage, indicated by the scanner, was 42% and 9%, respectively. The two spectra were normalized for the sum of counts in the scatter window centered at 118 keV (12% width), to allow for a visual comparison between them.

The spectrum acquired at 2.7 GBq presented deformations related to the high-count regime in which it was acquired, affected by increasing non-linear phenomena like the pulse pile-up effect and dead time. The ratio between mean counts in the main photopeak window (75–87 KeV) and in the scatter window (111–125 keV) is depicted in Fig. 2b, as the function of the total activity in the phantom. The resulting correlation coefficient between count ratio and activity at scanning time is −0.93 (*p value =* 1.94e^-7^), with a linear regression line of $y=2.1-\frac{0.09}{GBq}x$. This relation emphasizes the limitation of using a fixed k-factor, independent of ^166^Ho activity, to compensate for scatter.

The count rate curve in the 81-keV photopeak window is shown in Fig. 3. For both Symbia T16 and T2, the PDM model was fitted to the measured count rate data and forced to pass through the axis origin. Dead time influenced the ideally linear relation between activity and counts recovered (*C*(*A*) = *α A*), leading to a progressive count loss with increasing activity. The activity at which the 20% count loss was reported (*R*_20%_) was recovered at 714 MBq and 762 MBq for Symbia T2 and Symbia T16, respectively.

**Fig. 4:**
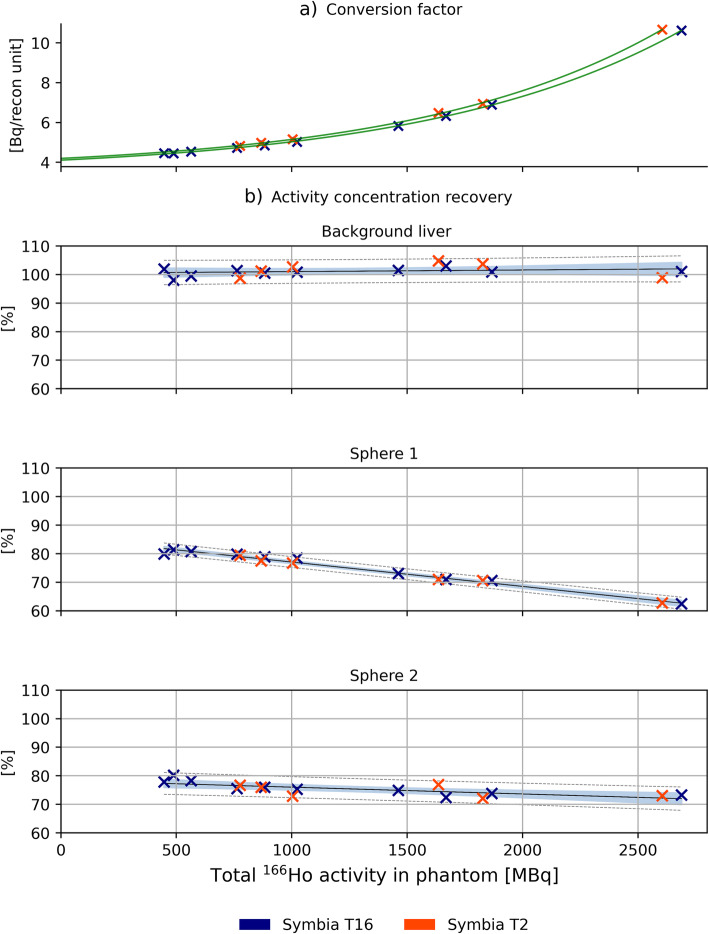
**a** Individual conversion factor between Bq and reconstruction values was computed for each measurement based on the total activity in the phantom at the scanning time. Its trend, as function of activity, is reported together with the individual measurements. **b** Activity concentration recovery as function of total activity in phantom at scanning time. A complete activity concentration recovery is obtained for non-tumorous liver (**b**)—top panel) while partial recovery for the spheres (**b**)—middle and bottom panel). Linear regression is represented by the black line, while gray dashed lines represent prediction intervals. Data in the blue area are within the confidence intervals

Conversion factors for reconstruction units to Bq, based on the known activity in the phantom at the time of scanning, are plotted in Fig. 4a, as a function of total activity. Activity concentration recovery as a function of total activity in the phantom is reported in Fig. 4b for the non-tumorous liver, S1 and S2. The non-tumorous liver compartment showed a non-significant (*p value* = 0.34) correlation with activity (*ρ* =0.256), with a mean ± standard deviation of 101.2 ± 1.8%. The activity concentration recoveries in the tumor compartments S1 and S2 were negatively correlated with activity (*ρ* =−0.97, *p value* = 2.28e^-10^, and *ρ* = –0.77, *p value* = 4.77e^-^^4^, respectively), with linear regression lines of $y=85.6\%\left(1-\frac{0.10}{GBq}x\ \right)$ and $y=78.4\%\left(1-\frac{0.03}{GBq}x\right)$.

No artifacts were reported in the images, when visually assessed by a nuclear medicine expert.

**Fig. 5:**
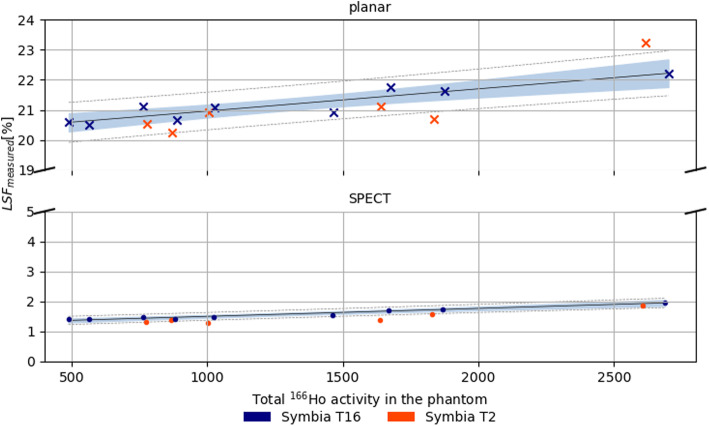
LSF_measured_ in the lungs based on planar images (top panel) and SPECT reconstructions (bottom panel). Linear regression is represented by the black line, while gray dashed lines represent prediction intervals. Data in the blue area are within the confidence intervals

LSF_measured_ is shown in Fig. 5 for both the planar-based approach (top panel) and SPECT-based approach (bottom panel). Despite the LSF_nominal_ was equal to 0, mean and standard deviations of the measured lung shunt fraction were 21.16% ± 0.55% for the planar-based method and 1.57% ± 0.19% for the SPECT-based method. The corresponding linear regression lines were $y=20.2\%\left(1-\frac{0.04}{GBq}\ x\right)$ and $y=1.2\%\left(1-\frac{0.25}{GBq}\ x\right)$. The correlation with activity was 0.85 (*p value =* 3.70e^-3^) and 0.95 (*p value* = 8.76e^-5^) for the planar- and SPECT-based method, respectively.

## Discussion

Count rate saturation and its effect on quantification are of importance for radiation peri-therapeutic dosimetry in nuclear medicine imaging. The impact of high activity on dead time and pulse pile-up was evident in the recorded energy spectra (Fig. 2a), where a relatively lower ^166^Ho photopeak was registered for the spectrum acquired during high-count rate, corresponding to a total activity in the phantom of 2.7 GBq. With increasing count rate, the amount of relative scatter is increasing, which is evident in Fig. 2b where the ratio between the mean of counts in the main photopeak and in the scatter window decreased with increasing activity. In our study, activity values corresponding to a 20% count loss were 714 MBq and 762 MBq for Symbia T2 and T16, respectively. In the work by Elschot et al. [4], a value for the A_linmax,_ defined as the highest activity with less than 2% loss of count rate, of approximately 1.5 GBq was reported. The difference between these findings and the values reported in the current work may be explained by the different gamma cameras employed, and the different methods used for the measurements. In the previous study, a Philips FORTE gamma camera was used. This camera operates a “high-count rate mode,” which is automatically activated at count rates above 20 kcps [13]. Moreover, to determine the A_linmax,_ an indirect approach was taken [4], which included the measurement of an intrinsic count rate curve (i.e., acquired without collimators) of an activity-filled vial in the full energy window, combined with separate measurements of the system sensitivity using a Petri dish with a thin layer of activity, hence without scatter material producing scattered photons and bremsstrahlung, in both the full-energy window and the photopeak window, underestimating the intrinsic count rate. Moreover, as can be appreciated from Fig. 2, dead-time effects become increasingly detrimental for a narrow photopeak window. In the present study, therefore, the focus was on mimicking patient geometry in a clinical scenario, directly measuring the number of counts in the main photopeak window for a range of activities.

This former study showed that the use of a single-conversion factor for quantification is insufficient for activities above several hundreds of MBq and that dead time effects need to be taken into account. The challenges of this process may be avoided altogether by scaling reconstructions to the known injected activity on a patient-to-patient basis, on the assumption that all of the activity is within the field of view. Using this scaling method, the activity concentration recovery in the liver compartment presented values close to 100%, regardless of total activity. This paves the way to a reliable assessment of absorbed dose in the healthy liver up to at least 2.7 GBq. This method may be used for dosimetric assessment of healthy liver absorbed dose, a decision driving parameter in radioembolization procedures.

Tumor dosimetry based on ^166^Ho SPECT/CT is challenging, especially when relying on standard commercially available reconstruction methods. These methods do not take into account the full emission spectrum and bremsstrahlung and typically rely on energy window-based scatter correction methods. The triple energy window method is inadequate for ^166^Ho due to the characteristic lead X-ray emission lines around 74 keV, just below the ^166^Ho photopeak. Reconstructions using dedicated Monte-Carlo methods significantly improve the image quality [14], but are typically not available for standard clinical use. Because of this, standard clinical ^166^Ho reconstructions are hampered by poor resolution due to a large partial volume effect and contain photopeak scatter and lead X-ray contamination.

At the high activity levels typically encountered after therapy, dead time effects become significant as well. Dead time effects add further issues to be considered when aiming at adequate dosimetry in the tumor compartments.

The impact of the dead time effect can be observed in the decrease of the activity concentration recovery for the spheres (representing tumors). Hence, at high ^166^Ho count rate, tumor dosimetry performed on clinically reconstructed SPECT scans is impaired even more.

The limitation due to the high-count regime is made clear when NEMA Image Quality (IQ) measurements are performed. The contrast recovery coefficients, for the nominal VOIs of the spheres, range from 9% for the smallest sphere (volume = 0.52 mL) to 47%, for the largest sphere (volume = 26.52 mL), showing the impact of the PVE. When the sphere VOI is dilated to correct for the PVE, using the same approach as presented for the anthropomorphic phantom used in this study, an increase in the contrast recovery coefficient is observed. However, this increase leads to a recovery of approximately 80%, which is in line with the activity concentration recovery obtained for the S1 and S2 spheres at lowest activities (see Fig. 4 b, middle and bottom panel), where the effect of the high-count regime was minor. This incomplete recovery is partly due to the suboptimal scatter correction method and was analyzed in details in the work by Elschot et al. [14] where several reconstruction methods were compared against one another for the computation of the contrast recovery coefficient, as function of NEMA IQ phantom sphere volumes.

Despite no activity was present in the lung compartment, LSF_nominal_ = 0, activity was measured in the lungs due to scatter. This phenomenon, quantified through LSF_measured_, using both planar and SPECT images, confirmed the superiority of SPECT images for lung shunt estimation [15, 16]. However, it is interesting to notice that, for both planar and SPECT images, LSF_measured_ was only slightly affected by the high-count rate.

The study was conducted under the assumption that all the injected activity was within the imaged field of view, and considering the measurement of residual activity a mandatory clinical practice. This allowed to the computation of the total injected activity in the patient to properly scale the reconstructed scans. This study has indeed showed that, at high ^166^Ho count rate, the scaling to the activity at scanning time plays a determinant role. However, for tumors VOI, where the activity concentration was higher, this approach was not sufficient to obtain a complete activity concentration recovery in the considered activity range, as it happened for the non-tumorous liver.

Some limitations apply to the study: images were acquired with a specific scanner; thus, the measurements reported in this study are vendor-specific. Moreover, administered activities for radioembolization procedures can be up to 10 GBq or more, while in this study the maximum total activity in the phantom was 2.7 GBq. In addition, the tumor to non-tumorous liver ratio, even though realistic, was fixed, and thus, it was not possible to assess gamma camera performance for different concentration ratios. Similarly, total tumor volume was limited to 40 mL (1200 mL total liver volume), whereas much larger volumes are often encountered. Moreover, during a patient scan, breathing artifacts and patient movement can play an important role. Notwithstanding this, an anthropomorphic phantom has the advantage to better mimic the average patient volume characteristics compared to the NEMA Image Quality phantom.

In the future, a similar experiment including breathing motion may be conducted, analyzing different possible methods to correct the consequent artifacts.

## Conclusions

^166^Ho quantitative reconstructions are deeply affected by the high-count regime, requiring a scaling to the total activity at the scanning time, making its accurate knowledge mandatory. If properly scaled, ^166^Ho reconstructions acquired up to 2.7 GBq provide reliable healthy liver dosimetry. However, tumor dosimetry remains inadequate, further challenged by the increasing dead time effect. At high-count rate, no image artifacts are reported. Measured lung shunt fractions were relatively constant, independent of total activity.

## Data Availability

The datasets used and/or analyzed during the current study are available from the corresponding author on reasonable request.

## Abbreviations

^166^Ho: Holmium-166
^90^Y: Yttrium-90
ACR: Activity concentration recovery
LDCT: Low-dose computed tomography
LSF: Lung shunt fraction
PDM: Paralyzable detector model
PVE: Partial volume effect
S1: Sphere 1
S2: Sphere 2
SPECT: Single photon emission computed tomography
VOI: Volume of interest
